# 2-(1*H*-Imidazol-1-yl)-4-[3-(trifluoro­meth­yl)phen­yl]-1,3-thia­zole

**DOI:** 10.1107/S1600536813000615

**Published:** 2013-01-16

**Authors:** Konstantin V. Kudryavtsev, Andrei V. Churakov, Jih-Hwa Guh

**Affiliations:** a Department of Chemistry, M.V. Lomonosov Moscow State University, Leninskie Gory 1/3, Moscow 119991, Russian Federation; b Institute of Physiologically Active Compounds, Russian Academy of Sciences, Chernogolovka 142432, Moscow Region, Russian Federation; c Institute of General and Inorganic Chemistry, Russian Academy of Sciences, Leninskii prosp. 31, Moscow 119991, Russian Federation; d School of Pharmacy, National Taiwan University, Taipei 100, Taiwan

## Abstract

The title compound, C_13_H_8_F_3_N_3_S, consists of three linked aromatic rings. The whole mol­ecule (except for the three F atoms) is planar to within 0.225 (2) Å. In the crystal, adjacent mol­ecules are linked into chains along the *ac* diagonal by weak C—H⋯N inter­actions.

## Related literature

For general background to the synthesis of imidazolo­thia­zoles by copper-catalysed coupling, see: Zhu *et al.* (2007 ▶).
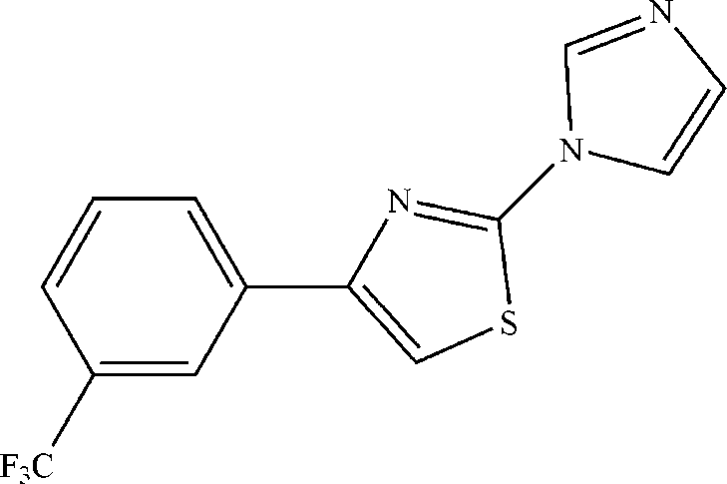


## Experimental

### 

#### Crystal data

C_13_H_8_F_3_N_3_S
*M* *_r_* = 295.28
Monoclinic, 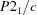

*a* = 8.4152 (7) Å
*b* = 19.2403 (15) Å
*c* = 8.4105 (7) Å
β = 114.210 (1)°
*V* = 1241.98 (18) Å^3^
*Z* = 4
Mo *K*α radiation
μ = 0.29 mm^−1^
*T* = 150 K
0.40 × 0.20 × 0.10 mm

#### Data collection

Bruker SMART APEXII diffractometer
Absorption correction: multi-scan (*SADABS*; Bruker, 2008 ▶) *T*_min_ = 0.893, *T*_max_ = 0.972
9797 measured reflections
2716 independent reflections
2164 reflections with *I* > 2σ(*I*)
*R*_int_ = 0.049

#### Refinement

*R*[*F* ^2^ > 2σ(*F* ^2^)] = 0.041
*wR*(*F* ^2^) = 0.103
*S* = 1.07
2716 reflections
213 parameters
All H-atom parameters refined
Δρ_max_ = 0.27 e Å^−3^
Δρ_min_ = −0.27 e Å^−3^

### 

Data collection: *APEX2* (Bruker, 2008 ▶); cell refinement: *SAINT* (Bruker, 2008 ▶); data reduction: *SAINT*; program(s) used to solve structure: *SHELXTL* (Sheldrick, 2008 ▶); program(s) used to refine structure: *SHELXTL*; molecular graphics: *SHELXTL*; software used to prepare material for publication: *SHELXTL*.

## Supplementary Material

Crystal structure: contains datablock(s) I, global. DOI: 10.1107/S1600536813000615/ff2095sup1.cif

Structure factors: contains datablock(s) I. DOI: 10.1107/S1600536813000615/ff2095Isup2.hkl

Supplementary material file. DOI: 10.1107/S1600536813000615/ff2095Isup3.cml

Additional supplementary materials:  crystallographic information; 3D view; checkCIF report

## Figures and Tables

**Table 1:** Hydrogen-bond geometry (Å, °)

*D*—H⋯*A*	*D*—H	H⋯*A*	*D*⋯*A*	*D*—H⋯*A*
C9—H9⋯N3^i^	0.95 (2)	2.34 (2)	3.278 (2)	169.4 (18)

Symmetry code: (i) 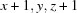.

**Fig. 1.**
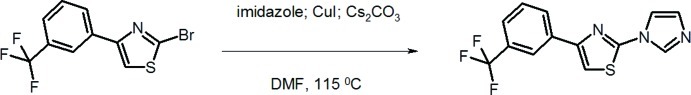
Reaction scheme.

**Fig. 2.**
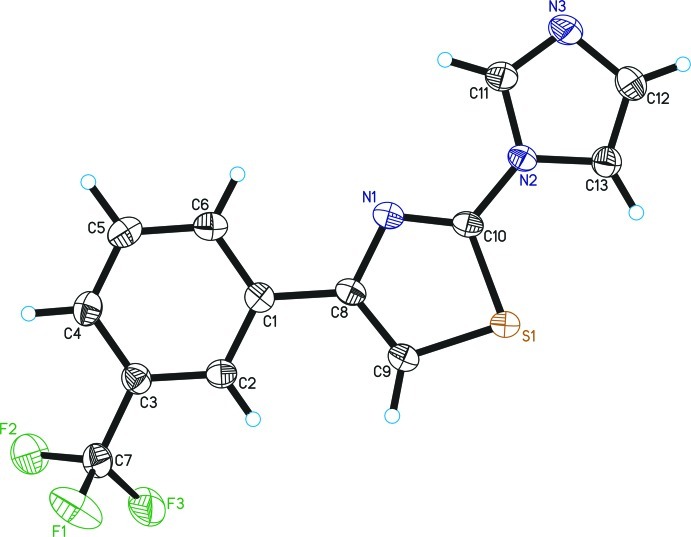
The molecular structure of the title compound, showing the numbering scheme adopted. Displacement ellipsoids are shown at the 50% probability level.

**Fig. 3.**
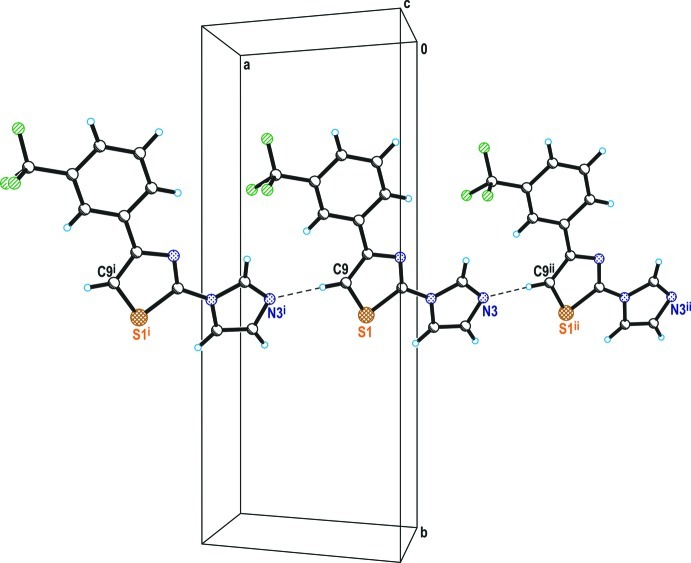
Chains along ac-diagonal in the structure of the title compound. C—H···N interactions are shown as dashed lines. [Symmetry codes: (i) 1 + x, y, 1 + z; (ii) -1 + x, y, -1 + z.]

## supplementary crystallographic information

### Comment

The title compound, C_13_H_8_F_3_N_3_S, which is a potential anticancer agent, consists of the three linked aromatic and heteroaromatic cycles, which were condensed by copper-catalyzed method (Fig. 1). The whole molecule (except three fluorine atoms) is planar within 0.225 (2) Å (Fig. 2).

In the crystal, the adjacent molecules are combined in chains along *ac*-diagonal by weak C—H···N interactions (Fig. 3). The C···N separation is equal to 3.278 (2) Å and C—H···N angle is close to linear (169 (2) °).

### Experimental

2-Bromo-4-(3-(trifluoromethyl)phenyl)thiazole (0.800 g, 2.60 mmol) was added to a stirred suspension of imidazole (0.345 g, 5.07 mmol), CuI (0.209 g, 1.10 mmol) and Cs_2_CO_3_ (1.830 g, 5.62 mmol) in 30 ml of DMF under argon atmosphere. The reaction mixture was stirred for 8 h at rt and then for 7 h at 115 °C. After cooling to the ambient temperature the reaction mixture was filtered and precipitate was washed with 30 ml of DMF. The solution was concentrated under vacuum and residue was diluted with 80 ml of ethyl acetate. Organic phase was washed with water (2 *x* 10 ml) and saturated solution of NH_4_Cl (1 *x* 10 ml), dried over Na_2_SO_4_, concentrated and purified by column chromatography on silica gel 60 (particle size 0.040–0.063 mm) using CHCl_3_—MeOH (gradient from 1:0 to 50:1) as eluent. 2-(1*H*-Imidazol-1-yl)-4-(3-(trifluoromethyl)phenyl)thiazole, yield 368 mg (48%), yellowish crystals, mp 99–100°C. ^1^H NMR (400 MHz, CDCl_3_/DMSO-d_6_ 5:1): *δ* 7.16 (s, 1H), 7.52–7.56 (m, 2H), 7.67 (s, 1H), 7.75 (s, 1H), 8.09–8.11 (m, 1H), 8.15 (s, 1H), 8.40 (s, 1H). ^13^C NMR (100 MHz, CDCl_3_/DMSO-d_6_ 5:1): *δ* 111.88, 118.24, 122.95, 122.98, 125.00, 125.03, 129.59, 129.66, 130.17, 130.62, 134.35, 135.63, 150.89, 157.05. Found, %: C, 52.95; H, 2.69; N, 14.20. C_13_H_8_F_3_N_3_S. Calculated, %: C, 52.88; H, 2.73; N, 14.23. The crystals were obtained by slow evaporation of the CDCl_3_/DMSO-d_6_ (5:1) solution.

### Refinement

All hydrogen atoms were located in a difference Fourier map and refined with isotropic thermal parameters.

### Crystal data

**Table:**

C_13_H_8_F_3_N_3_S	*F*(000) = 600
*M**_r_* = 295.28	*D*_x_ = 1.579 Mg m^−^^3^
Monoclinic, *P*2_1_/*c*	Mo *K*α radiation, λ = 0.71073 Å
Hall symbol: -P 2ybc	Cell parameters from 1717 reflections
*a* = 8.4152 (7) Å	θ = 3.1–24.5°
*b* = 19.2403 (15) Å	µ = 0.29 mm^−^^1^
*c* = 8.4105 (7) Å	*T* = 150 K
β = 114.210 (1)°	Block, colourless
*V* = 1241.98 (18) Å^3^	0.40 × 0.20 × 0.10 mm
*Z* = 4	

### Data collection

**Table:**

Bruker SMART APEXII diffractometer	2716 independent reflections
Radiation source: fine-focus sealed tube	2164 reflections with *I* > 2σ(*I*)
Graphite monochromator	*R*_int_ = 0.049
ω scans	θ_max_ = 27.0°, θ_min_ = 2.1°
Absorption correction: multi-scan (*SADABS*; Bruker, 2008)	*h* = −10→9
*T*_min_ = 0.893, *T*_max_ = 0.972	*k* = −24→24
9797 measured reflections	*l* = −10→10

### Refinement

**Table:**

Refinement on *F*^2^	Primary atom site location: structure-invariant direct methods
Least-squares matrix: full	Secondary atom site location: difference Fourier map
*R*[*F*^2^ > 2σ(*F*^2^)] = 0.041	Hydrogen site location: difference Fourier map
*wR*(*F*^2^) = 0.103	All H-atom parameters refined
*S* = 1.07	*w* = 1/[σ^2^(*F*_o_^2^) + (0.0429*P*)^2^ + 0.2754*P*] where *P* = (*F*_o_^2^ + 2*F*_c_^2^)/3
2716 reflections	(Δ/σ)_max_ < 0.001
213 parameters	Δρ_max_ = 0.27 e Å^−^^3^
0 restraints	Δρ_min_ = −0.27 e Å^−^^3^

### Special details

**Table:**

Geometry. All e.s.d.'s (except the e.s.d. in the dihedral angle between two l.s. planes) are estimated using the full covariance matrix. The cell e.s.d.'s are taken into account individually in the estimation of e.s.d.'s in distances, angles and torsion angles; correlations between e.s.d.'s in cell parameters are only used when they are defined by crystal symmetry. An approximate (isotropic) treatment of cell e.s.d.'s is used for estimating e.s.d.'s involving l.s. planes.
Refinement. Refinement of *F*^2^ against ALL reflections. The weighted *R*-factor *wR* and goodness of fit *S* are based on *F*^2^, conventional *R*-factors *R* are based on *F*, with *F* set to zero for negative *F*^2^. The threshold expression of *F*^2^ > σ(*F*^2^) is used only for calculating *R*-factors(gt) *etc*. and is not relevant to the choice of reflections for refinement. *R*-factors based on *F*^2^ are statistically about twice as large as those based on *F*, and *R*- factors based on ALL data will be even larger.

### Fractional atomic coordinates and isotropic or equivalent isotropic displacement parameters (Å^2^)

**Table:**

	*x*	*y*	*z*	*U*_iso_*/*U*_eq_	
S1	0.22949 (6)	0.55985 (2)	0.40649 (6)	0.02492 (15)	
N1	0.0577 (2)	0.44567 (7)	0.36963 (19)	0.0214 (3)	
N2	−0.0844 (2)	0.52744 (7)	0.14612 (18)	0.0207 (3)	
N3	−0.3431 (2)	0.52646 (9)	−0.0761 (2)	0.0306 (4)	
F1	0.62622 (19)	0.32402 (8)	1.15343 (17)	0.0571 (4)	
F2	0.64762 (19)	0.22590 (6)	1.0452 (2)	0.0571 (4)	
F3	0.74065 (16)	0.31841 (7)	0.97113 (19)	0.0530 (4)	
C1	0.2432 (2)	0.37530 (9)	0.6250 (2)	0.0215 (4)	
C2	0.4056 (3)	0.36375 (10)	0.7611 (2)	0.0234 (4)	
C3	0.4358 (3)	0.30371 (9)	0.8600 (2)	0.0250 (4)	
C4	0.3055 (3)	0.25486 (10)	0.8277 (3)	0.0277 (4)	
C5	0.1440 (3)	0.26582 (10)	0.6924 (3)	0.0288 (5)	
C6	0.1129 (3)	0.32528 (10)	0.5908 (3)	0.0256 (4)	
C7	0.6105 (3)	0.29265 (10)	1.0058 (3)	0.0331 (5)	
C8	0.2102 (2)	0.43953 (9)	0.5201 (2)	0.0204 (4)	
C9	0.3176 (3)	0.49573 (9)	0.5588 (2)	0.0240 (4)	
C10	0.0540 (2)	0.50577 (9)	0.2999 (2)	0.0205 (4)	
C11	−0.2399 (3)	0.49374 (10)	0.0647 (2)	0.0285 (5)	
C12	−0.2503 (3)	0.58436 (11)	−0.0854 (3)	0.0286 (4)	
C13	−0.0922 (3)	0.58620 (10)	0.0485 (2)	0.0266 (4)	
H9	0.425 (3)	0.5042 (10)	0.656 (3)	0.028 (5)*	
H6	0.004 (3)	0.3331 (10)	0.496 (3)	0.028 (5)*	
H2	0.489 (3)	0.3964 (11)	0.783 (3)	0.031 (6)*	
H11	−0.263 (3)	0.4517 (11)	0.113 (3)	0.035 (6)*	
H5	0.053 (3)	0.2292 (11)	0.670 (3)	0.040 (6)*	
H4	0.328 (3)	0.2144 (11)	0.900 (3)	0.037 (6)*	
H12	−0.298 (3)	0.6159 (12)	−0.176 (3)	0.043 (7)*	
H13	0.002 (3)	0.6192 (12)	0.080 (3)	0.044 (7)*	

### Atomic displacement parameters (Å^2^)

**Table:**

	*U*^11^	*U*^22^	*U*^33^	*U*^12^	*U*^13^	*U*^23^
S1	0.0198 (3)	0.0248 (2)	0.0255 (3)	−0.00220 (19)	0.00454 (19)	0.00161 (18)
N1	0.0189 (8)	0.0255 (8)	0.0196 (8)	0.0012 (6)	0.0077 (6)	−0.0024 (6)
N2	0.0192 (8)	0.0238 (7)	0.0172 (7)	0.0011 (6)	0.0055 (6)	−0.0012 (6)
N3	0.0268 (9)	0.0364 (9)	0.0214 (8)	−0.0014 (7)	0.0028 (7)	0.0014 (7)
F1	0.0494 (9)	0.0731 (10)	0.0318 (7)	0.0124 (8)	−0.0007 (6)	−0.0037 (7)
F2	0.0446 (9)	0.0321 (7)	0.0713 (10)	0.0094 (6)	0.0003 (7)	0.0201 (6)
F3	0.0223 (7)	0.0689 (10)	0.0589 (9)	0.0053 (6)	0.0076 (6)	0.0279 (7)
C1	0.0230 (10)	0.0223 (9)	0.0209 (9)	0.0020 (7)	0.0107 (8)	−0.0022 (7)
C2	0.0210 (10)	0.0251 (9)	0.0244 (10)	0.0001 (8)	0.0096 (8)	−0.0013 (7)
C3	0.0256 (11)	0.0233 (9)	0.0250 (10)	0.0028 (8)	0.0094 (8)	0.0007 (7)
C4	0.0315 (11)	0.0222 (9)	0.0319 (11)	0.0007 (8)	0.0155 (9)	0.0025 (8)
C5	0.0257 (11)	0.0260 (10)	0.0361 (11)	−0.0043 (8)	0.0142 (9)	−0.0040 (8)
C6	0.0214 (10)	0.0273 (10)	0.0270 (10)	−0.0003 (8)	0.0088 (8)	−0.0046 (8)
C7	0.0315 (12)	0.0276 (10)	0.0357 (12)	0.0029 (9)	0.0092 (9)	0.0084 (8)
C8	0.0176 (9)	0.0250 (9)	0.0186 (9)	0.0028 (7)	0.0074 (7)	−0.0018 (7)
C9	0.0198 (10)	0.0267 (10)	0.0229 (9)	0.0014 (8)	0.0059 (8)	0.0008 (7)
C10	0.0179 (9)	0.0257 (9)	0.0176 (9)	0.0005 (7)	0.0068 (7)	−0.0029 (7)
C11	0.0274 (11)	0.0297 (10)	0.0220 (10)	−0.0044 (8)	0.0037 (8)	−0.0012 (8)
C12	0.0307 (11)	0.0316 (10)	0.0224 (10)	0.0043 (9)	0.0099 (8)	0.0034 (8)
C13	0.0259 (11)	0.0282 (10)	0.0255 (10)	−0.0005 (8)	0.0104 (8)	0.0036 (8)

### Geometric parameters (Å, º)

**Table:**

S1—C9	1.7128 (19)	C2—C3	1.385 (3)
S1—C10	1.7266 (18)	C2—H2	0.90 (2)
N1—C10	1.291 (2)	C3—C4	1.384 (3)
N1—C8	1.390 (2)	C3—C7	1.494 (3)
N2—C11	1.367 (2)	C4—C5	1.385 (3)
N2—C13	1.383 (2)	C4—H4	0.96 (2)
N2—C10	1.403 (2)	C5—C6	1.387 (3)
N3—C11	1.307 (2)	C5—H5	1.00 (2)
N3—C12	1.381 (3)	C6—H6	0.95 (2)
F1—C7	1.337 (3)	C8—C9	1.360 (3)
F2—C7	1.331 (2)	C9—H9	0.95 (2)
F3—C7	1.339 (3)	C11—H11	0.96 (2)
C1—C2	1.394 (3)	C12—C13	1.346 (3)
C1—C6	1.397 (3)	C12—H12	0.93 (2)
C1—C8	1.477 (2)	C13—H13	0.96 (2)
			
C9—S1—C10	88.27 (9)	F2—C7—F1	106.30 (17)
C10—N1—C8	109.34 (15)	F2—C7—F3	106.36 (18)
C11—N2—C13	106.72 (15)	F1—C7—F3	104.87 (18)
C11—N2—C10	125.71 (16)	F2—C7—C3	113.16 (17)
C13—N2—C10	127.57 (16)	F1—C7—C3	112.79 (18)
C11—N3—C12	105.01 (17)	F3—C7—C3	112.71 (17)
C2—C1—C6	118.77 (17)	C9—C8—N1	115.14 (16)
C2—C1—C8	120.30 (17)	C9—C8—C1	125.36 (16)
C6—C1—C8	120.93 (17)	N1—C8—C1	119.50 (16)
C3—C2—C1	120.20 (18)	C8—C9—S1	110.65 (14)
C3—C2—H2	121.4 (13)	C8—C9—H9	130.2 (12)
C1—C2—H2	118.4 (13)	S1—C9—H9	119.0 (12)
C4—C3—C2	120.85 (18)	N1—C10—N2	122.77 (16)
C4—C3—C7	119.88 (17)	N1—C10—S1	116.59 (13)
C2—C3—C7	119.25 (17)	N2—C10—S1	120.64 (13)
C3—C4—C5	119.30 (18)	N3—C11—N2	111.68 (18)
C3—C4—H4	119.6 (14)	N3—C11—H11	128.1 (13)
C5—C4—H4	121.1 (14)	N2—C11—H11	120.2 (13)
C4—C5—C6	120.36 (19)	C13—C12—N3	111.19 (17)
C4—C5—H5	117.8 (13)	C13—C12—H12	128.0 (14)
C6—C5—H5	121.8 (13)	N3—C12—H12	120.8 (14)
C5—C6—C1	120.50 (18)	C12—C13—N2	105.38 (17)
C5—C6—H6	121.5 (12)	C12—C13—H13	131.8 (14)
C1—C6—H6	118.0 (12)	N2—C13—H13	122.8 (14)
			
C6—C1—C2—C3	−0.1 (3)	C2—C1—C8—N1	170.46 (17)
C8—C1—C2—C3	179.52 (17)	C6—C1—C8—N1	−9.9 (3)
C1—C2—C3—C4	−1.0 (3)	N1—C8—C9—S1	0.5 (2)
C1—C2—C3—C7	−179.66 (18)	C1—C8—C9—S1	−178.79 (15)
C2—C3—C4—C5	1.2 (3)	C10—S1—C9—C8	−0.46 (15)
C7—C3—C4—C5	179.87 (19)	C8—N1—C10—N2	−179.81 (16)
C3—C4—C5—C6	−0.3 (3)	C8—N1—C10—S1	−0.2 (2)
C4—C5—C6—C1	−0.8 (3)	C11—N2—C10—N1	9.5 (3)
C2—C1—C6—C5	1.0 (3)	C13—N2—C10—N1	−171.01 (18)
C8—C1—C6—C5	−178.61 (18)	C11—N2—C10—S1	−170.05 (15)
C4—C3—C7—F2	26.1 (3)	C13—N2—C10—S1	9.4 (3)
C2—C3—C7—F2	−155.26 (19)	C9—S1—C10—N1	0.41 (16)
C4—C3—C7—F1	−94.6 (2)	C9—S1—C10—N2	180.00 (16)
C2—C3—C7—F1	84.0 (2)	C12—N3—C11—N2	−0.4 (2)
C4—C3—C7—F3	146.84 (19)	C13—N2—C11—N3	0.4 (2)
C2—C3—C7—F3	−34.5 (3)	C10—N2—C11—N3	180.00 (17)
C10—N1—C8—C9	−0.2 (2)	C11—N3—C12—C13	0.2 (2)
C10—N1—C8—C1	179.13 (16)	N3—C12—C13—N2	0.0 (2)
C2—C1—C8—C9	−10.3 (3)	C11—N2—C13—C12	−0.3 (2)
C6—C1—C8—C9	169.30 (18)	C10—N2—C13—C12	−179.81 (18)

### Hydrogen-bond geometry (Å, º)

**Table:**

*D*—H···*A*	*D*—H	H···*A*	*D*···*A*	*D*—H···*A*
C9—H9···N3^i^	0.95 (2)	2.34 (2)	3.278 (2)	169.4 (18)

Symmetry code: (i) *x*+1, *y*, *z*+1.
